# 774 A Novel Facial Mask Design: A Single Case Report

**DOI:** 10.1093/jbcr/irae036.315

**Published:** 2024-04-17

**Authors:** Andria Martinez, Renée Warthman, Whitney Pirsig, Derek Murray, Karen J Richey, Kevin N Foster

**Affiliations:** Arizona Burn Center at Valleywise Health, Phoenix, AZ; Valleywise Health Medical Center - AZ Burn Center, Gilbert, AZ; Valleywise, Phoenix, AZ; Arizona Burn Center at Valleywise Health, Phoenix, AZ; Valleywise Health Medical Center - AZ Burn Center, Gilbert, AZ; Valleywise, Phoenix, AZ; Arizona Burn Center at Valleywise Health, Phoenix, AZ; Valleywise Health Medical Center - AZ Burn Center, Gilbert, AZ; Valleywise, Phoenix, AZ; Arizona Burn Center at Valleywise Health, Phoenix, AZ; Valleywise Health Medical Center - AZ Burn Center, Gilbert, AZ; Valleywise, Phoenix, AZ; Arizona Burn Center at Valleywise Health, Phoenix, AZ; Valleywise Health Medical Center - AZ Burn Center, Gilbert, AZ; Valleywise, Phoenix, AZ; Arizona Burn Center at Valleywise Health, Phoenix, AZ; Valleywise Health Medical Center - AZ Burn Center, Gilbert, AZ; Valleywise, Phoenix, AZ

## Abstract

**Introduction:**

Facial scarring can impact an individual’s self-worth and confidence. Treating facial scarring often includes fabrication and fitting of custom face masks. Facial masks are generally fabricated utilizing high or low temperature thermoplastic material. Facial masks may also include a silicone component to address scar formation. At present time, facial mask fabrication has evolved from plaster molding directly on the patient to 3-D scanning and subsequent molding. Impediments to current practice may include cost, delayed prescription, and breakage which can allow tissues to change which ultimately require extended modifications. This is a report of a single patient in which a 50/50 putty facemask with a thermoplastic overlay was utilized to address facial scarring, compliance, cost, and distance from clinic.

**Methods:**

This is a case study of a 5-year-old child who suffered deep partial thickness to full thickness burn injuries to his face for whom we created a facemask with a superimposed thermoplastic overlay to maintain the position of the 50/50 putty mask. The 50/50 facemask was fabricated in a t-shape with clearance of eyes, nose and mouth. The thermoplastic overlay was fabricated to contour the exact shape of the outer face over the 50/50 putty. The Patient and Observer Scar Assessment Scale (POSAS) was administered prior to applying the custom facemask and at day 42 to evaluate efficacy. Each item on the POSAS scale is scored from 1 to 10, with 1 being normal skin and 10 representing the worst scar possible. The total score can range from 6 to 60. There is a separate overall opinion score that is rated on the same 1-10 scale. The father completed the patient portion of the scale with feedback from the child.

**Results:**

The custom facemask was worn 23 hours per day for 42 days. The baseline patient score was 42 and the patient overall opinion of scarring score was 7. The observer score was 43 with an overall opinion of scarring score of 7. At day 42 both the patient score and patient overall opinion score had improved, 18 and 4 respectively. The observer score and observer overall opinion score also improved, 21 and 3 respectively.

**Conclusions:**

Utilization of a 50/50 putty facemask allowed for continuous wear with less incidence of modifications due to material composition. Utilization of a 50/50 putty facial mask accommodated tissue modulation without risk of erythema in a way that thermoplastic material is unable to achieve. The facemask utilized in this single case study provided standard of care treatment while increasing ease of management for patient, decreasing cost, and allowed for same day use following initial fabrication.

**Applicability of Research to Practice:**

Utilization of this type of facial mask whether exclusively or as an adjunct to a thermoplastic mask, may provide standard of care treatment and expedite scar management.

